# ZIF-8 Nanoparticle: A Valuable Tool for Improving Gene Delivery in Sperm-Mediated Gene Transfer

**DOI:** 10.1186/s12575-024-00229-2

**Published:** 2024-01-26

**Authors:** Marzieh Sameni, Parisa Moradbeigi, Sara Hosseini, Sayyed Mohammad Hossein Ghaderian, Vahid Jajarmi, Amir Hossein Miladipour, Hojat Basati, Maryam Abbasi, Mohammad Salehi

**Affiliations:** 1 Department of Medical Biotechnology, School of Advanced Technologies in Medicine, Shahid Beheshti University of Medical Sciences, Tehran, Iran; 2 Department of Clinical Sciences, School of Veterinary Medicine, Shiraz University, Shiraz, Iran; 3 Cellular and Molecular Biology Research Center, Shahid Beheshti University of Medical Sciences, Tehran, Iran; 4 Department of Medical Genetics, Shahid Beheshti University of Medical Sciences, Tehran, Iran; 5 Department of Nephrology, Clinical Research and Development Center at Shahid Modarres Hospital, Shahid Beheshti University of Medical Sciences, Tehran, Iran; 6 Tissue Engineering Department, TISSUEHUB Co, Tehran, Iran; 7 Department of Chemical Engineering, Faculty of Engineering, Tehran University, Tehran, Iran; 8 Department of Biology, Science and Research Branch, Islamic Azad University, Tehran, Iran; 9 Zhino-Gene Research Services Co, Tehran, Iran; 10 Hasti Noavaran Gene Royan, Tehran, Iran

**Keywords:** MOFs, ZIF-8, SMGT, Gene delivery, DNA Uptake

## Abstract

Metal–organic frameworks (MOFs) are porous materials with unique characteristics that make them well-suited for drug delivery and gene therapy applications. Among the MOFs, zeolitic imidazolate framework-8 (ZIF-8) has emerged as a promising candidate for delivering exogenous DNA into cells. However, the potential of ZIF-8 as a vector for sperm-mediated gene transfer (SMGT) has not yet been thoroughly explored.

This investigation aimed to explore the potential of ZIF-8 as a vector for enhancing genetic transfer and transgenesis rates by delivering exogenous DNA into sperm cells. To test this hypothesis, we employed ZIF-8 to deliver a plasmid expressing green fluorescent protein (GFP) into mouse sperm cells and evaluated the efficiency of DNA uptake. Our findings demonstrate that ZIF-8 can efficiently load and deliver exogenous DNA into mouse sperm cells, increasing GFP expression in vitro. These results suggest that ZIF-8 is a valuable tool for enhancing genetic transfer in SMGT, with important implications for developing genetically modified animals for research and commercial purposes. Additionally, our study highlights the potential of ZIF-8 as a novel class of vectors for gene delivery in reproductive biology.

Overall, our study provides a foundation for further research into using ZIF-8 and other MOFs as gene delivery systems in reproductive biology and underscores the potential of these materials as promising vectors for gene therapy and drug delivery.

**Supplementary Information:**

The online version contains supplementary material available at 10.1186/s12575-024-00229-2.

## Background

Sperm cells exhibit a unique and distinct structure characterized by their compactness, elongated flagellum, highly condensed chromatin, and limited cytoplasm, differentiating them from all other cell types. This unusual morphology has led to the perception that sperm cells are metabolically inactive [1, 2]. However, it is now widely recognized that sperm cells are highly specialized and terminal cells, possessing a densely compacted haploid genome within their nucleus. The compaction results from protamine binding to DNA, rendering sperm cells dormant and incapable of transcription and translation. Despite this, various experiments have been conducted to explore the capacity of sperm cells to serve as a vector for exogenous nucleic acid (ENA) [3].

The sperm-mediated gene transfer (SMGT) method has been employed in transgenesis across a range of species, as indicated by prior research [1, 4, 5]. SMGT holds promise as a potentially cost-effective and efficient approach for generating transgenic animals, thereby significantly augmenting their value in biomedical studies and commercial utilization. Nevertheless, additional enhancements are required to boost its effectiveness [6, 7].

The conventional methods of SMGT face a challenge due to inadequate rates of DNA uptake, as they depend on the sperm cells' spontaneous uptake of foreign DNA during the incubation process [7, 8]. In SMGT, exogenous DNA is spontaneously integrated into sperm cells, which function as a "natural transfection vector." This process involves the transfer of the genetic construct into the oocyte during in vitro fertilization (IVF) [9, 10].

Numerous studies have proposed techniques to enhance DNA uptake by sperm cells, such as electroporation [11, 12] and DMSO/DNA complex [13], although the offspring success rate remained limited [2]. Nevertheless, the use of nanoparticle nanoparticles for introducing foreign DNA into eukaryotic cells has demonstrated promising outcomes [14].

Nanoparticle-mediated delivery is a potentially valuable approach in reproductive biology. The unique structure and functional significance of reproductive tissues and gametes necessitate using minimally invasive research techniques that do not impede fertility or impact the development of subsequent offspring. Nanotechnology can potentially enhance the biological safety and effectiveness of numerous existing experimental methods [15, 16]. Notably, the flexible nature of nano-carriers greatly simplifies their use as delivery systems for various biological compounds. Metal–organic frameworks (MOFs) represent a class of porous materials with metallic centers connected by organic linkers [17–19]. They have been widely applied in diverse areas such as biomedicine [20–23], biotechnology [24–26]., and analytical chemistry [27, 28]. MOFs can be utilized for nucleic acid loading through various mechanisms, such as metal-phosphate coordination interaction [28], multivalent coordination between the DNA backbone phosphate and unsaturated zirconium sites present in MOFs [29], or encapsulation [30].

The modular technique employed in MOF synthesis permits accurate regulation of their physical characteristics and chemical properties [31]. Their porous structure and ability to adjust pore size and shape make MOFs ideal vehicles for drug loading. Consequently, they are considered as a prospective option for drug delivery applications [32, 33].

Zeolitic Imidazolate Frameworks (ZIFs), a particular classification of MOFs, are constructed through self-assembly of M-IM-M structures, where M denotes tetrahedrally-coordinated metal ions; these metal ions include cobalt (Co), copper (Cu), and zinc (Zn), while IM represents the imidazolate ligand [34]. The M-IM-M structure, which is oriented at an angle of 145°, plays a crucial role in establishing a zeolite-like topology [35, 36].

Zinc-based MOFs have been identified as potential nano-carriers since zinc ions exhibit low toxicity; ZIF-8 has been utilized for the delivery of various anticancer drugs, including curcumin (CCM) [37] and doxorubicin (DOX) [38, 39], camptothecin (CPT) [40], and CpG (cytosine, guanine, and phosphate) oligodeoxynucleotides [41]. Furthermore, the imidazole linkers are acknowledged for their remarkable pH-buffering capability, which is hypothesized to provide an increased potential to evade the endocytic pathway [42, 43]. The current investigation aims to assess the potential of MOFs, specifically ZIF-8, as an innovative approach for transporting exogenous DNA into sperm cells. We hypothesize that the unique porous structure of ZIF-8 could facilitate the DNA uptake by sperm cells, thereby potentially leading to increased genetic transfer and transgenesis rates. For this purpose, we conducted a series of experiments utilizing ZIF-8 to deliver a GFP-expressing plasmid into mouse sperm. Our findings demonstrate that ZIF-8 can effectively deliver exogenous DNA into mouse sperm cells, resulting in elevated levels of GFP expression in vitro. These results suggest that ZIF-8 holds promise as a valuable tool for enhancing genetic transfer in sperm-mediated gene transfer (SMGT), which could lead to the development of modified animals for research and commercial purposes.

## Materials and Methods

### Overview of the Study Design

**Fig. 1:**
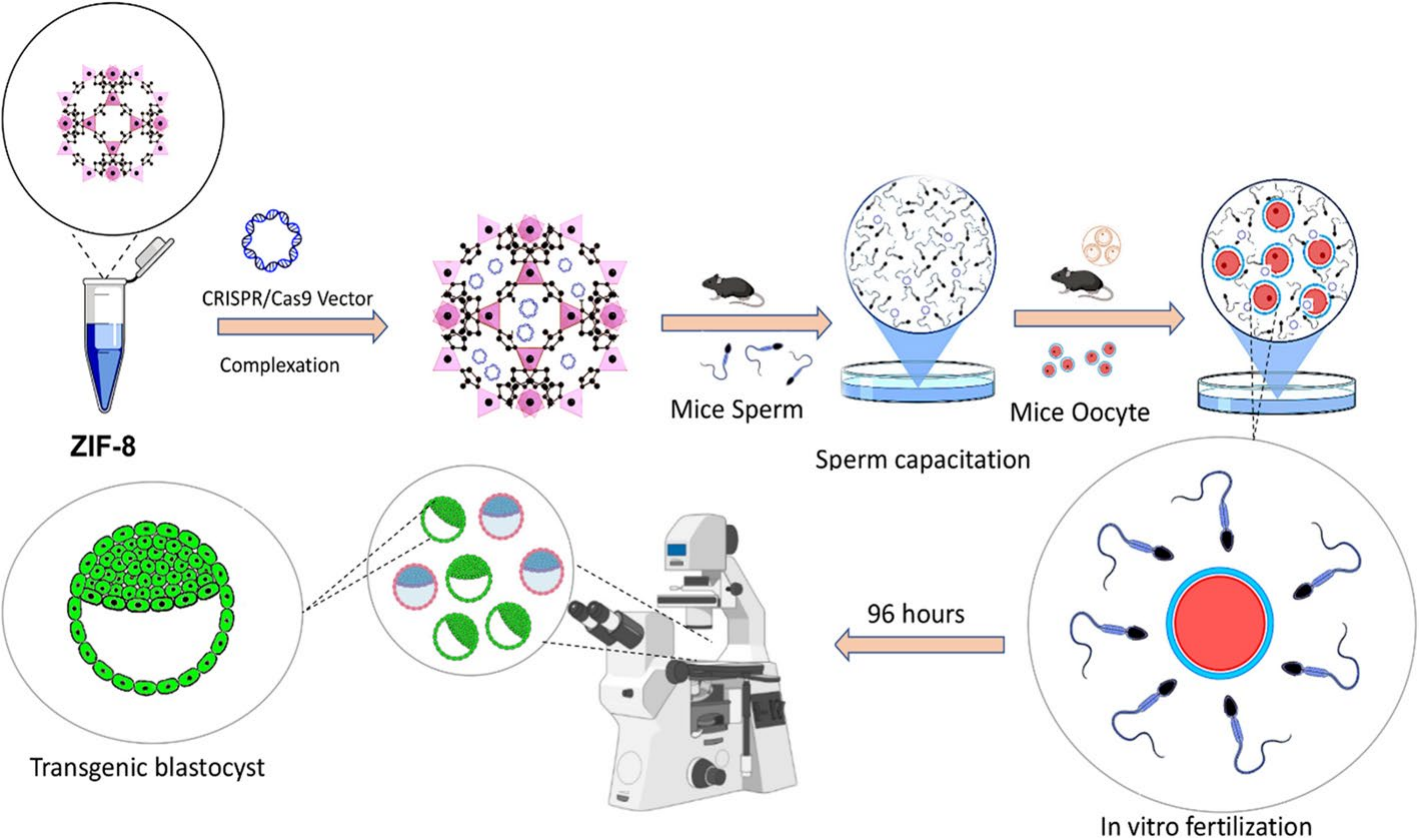
Schematic workflow of the research process. This figure shows the workflow of the research process, including the different steps involved in conducting a scientific study: Production of transgenic blastocyst through sperm-mediated gene transfer using ZIF-8 nano-carriers

This research aimed to explore the role of ZIF-8 in the gene uptake process in mouse spermatozoa. Figure 1 depicts the comprehensive steps involved in the process. Here, healthy male mice were used to collect spermatozoa via standard methods. The spermatozoa were then isolated from other cell types using density gradient centrifugation. Three groups were formed; one group served as a control, while the other group was treated with different concentrations of ZIF-8 to determine if ZIF-8 plays a role in gene uptake and DMSO as a positive control. The sperm cells were subjected to incubation while either including or excluding the presence of the ZIF-8 substance, and gene uptake was quantified using qPCR or other appropriate techniques.

Further analysis was conducted by measuring the viability, motility, and acrosomal reaction of spermatozoa using different staining, and the effect of ZIF-8 on embryo development was evaluated. Statistical tests of ANOVA were used to analyze the data and determine if there was a significant difference in gene uptake between the control and treated groups. The findings of the study were interpreted in the context of the hypothesis. The study design also considered ethical considerations, including animal welfare and adherence to relevant regulations. The study results offer valuable insights into the potential involvement of ZIF-8 in gene uptake in mouse spermatozoa, which could inform future research in this area. Overall, this research enhances our comprehension of the intricate mechanisms involved in the gene uptake in spermatozoa, which has important implications for reproductive biology and gene therapy.

### ZIF-8 synthesis

To synthesize nanoparticles, initially, 585g of Zn(NO3)26H2O (Sigma-Aldrich) were dissolved in 4 ml of deionized water. Then, 35.11g of 2-methylimidazole (obtained from Sigma-Aldrich) was dissolved in an additional 40 ml of deionized water. Subsequently, the zinc nitrate solution was combined with the 2-methylimidazole solution while being gently stirred for 30 min at room temperature. The resulting solution turned milky immediately after the two solutions were integrated. The synthesized solution was stirred for 24 h to ensure a complete reaction. Then, it was centrifuged at 4000 rpm for 15 min to remove any unreacted materials through washing. Finally, the settled product was dried at 65 degrees Celsius for 24 h to produce ZIF-8 [44]. The working solution was obtained by dissolving 1 mg of the synthesized powder in 1 mL of cell culture water. The solution was then sonicated for 30 min to obtain a homogeneous solution. Additionally, the solution was filtered using a 0.22-µm filter before incubating the sperm.

### Analytical Methods for Characterization of ZIF-8

To examine the morphology of ZIF-8 nanoparticles a scanning electron microscope (SEM) (device model: SEM FEI Quanta 200) was utilized. The nanoparticles were coated with gold under vacuum and analyzed using SEM. Fourier transform infrared spectrometry was conducted using a JASCO FT/IR-4600 device to determine functional groups. The infrared spectrum was measured within the range of 400 to 4000 cm^−1^ to determine the vibrations of ZIF-8. To examine the profile of ZIF-8 nanoparticle size distribution, Dynamic Light Scattering (DLS) analysis (DLS-nanoPartica (Model SZ-100 series) HORIBA manufacturing company), which is a physical method, was employed. This method determines the hydrodynamic sizes of nanoparticles and indicates the diameter of a nanoparticle in a state with movements, including those resulting from Brownian movements. The basis of this method is the interaction of light with particles. X-ray diffraction (XRD) analysis was performed using a Philips PW 1730 device manufactured by Philips to investigate the structure of nanoparticles.

### Statement of Ethics

All animal welfare and research procedures followed the guidelines in the Guide for the Care and Use of Laboratory Animals of the National Institutes of Health. The Institutional Animal Care and Research Ethics Committee of Shahid Beheshti University of Medical Sciences in Iran approved the guidelines. The experiments involving animals were conducted under the supervision of trained operators who followed strict protocols to ensure the mice were handled with utmost care and precision. Every possible measure was taken to minimize any potential distress or discomfort experienced by the animals.

### Animals

The mice used in this experimental investigation were procured from the Pasteur Institute and were housed in an environment with a controlled temperature range and humidity that ranged from 40 to 70%. The room was illuminated for 12 h and subsequently covered in darkness. The mice were provided with unlimited availability of both water and food. The animals were treated humanely and ethically throughout the study to reduce their discomfort. The experimental animals' housing, maintenance, and use were carried out in adherence to the ethical principles for conducting animal research by following the guidelines outlined in the National Institutes of Health's Guide for the Care and Use of Laboratory Animals. These guidelines guarantee adherence to the established ethical standards in animal research.

### Sperm Collection

Male BDF1 mice aged between 8 to 10 weeks old were used in this study. The reproductive organs of these mice were surgically removed after cervical dislocation. To collect the sperm, the cauda epididymis was retrieved at room temperature and placed in a culture medium that had been pre-warmed. Subsequently, under the observation of a stereomicroscope, the cauda epididymis was gently dissected along its length to release the sperm cells. The sperm cells were subsequently gathered within a human tube fluid (HTF) solution at room temperature.

### Preparation and incubation of spermatozoa with different concentrations of ZIF-8

Several ratios of ZIF-8 were prepared from a stock solution with a concentration of 1mg mL^−1^. The spermatozoa samples were then subjected to an incubation process, in which different concentrations of ZIF-8 were introduced to examine any potential effects on sperm motility and viability. Specifically, the concentrations of ZIF-8 utilized were 7.5 µg mL^−1^, 15 µg mL^−1^, 30 µg mL^−1^, and 60 µg mL^−1^,90 µg mL^−1^,120 µg mL^−1^,150 µg mL^−1^, and 200 µg mL^−1^ compared to utilizing DMSO (3%) as a positive control and employing a control (sperm incubated in HTF) The incubation process was conducted for 30 min under controlled environmental conditions of temperature and humidity.

### Preparation of exogenous DNA

The pCAG-eCAS9-GFP-U6-gRNA, which included the enhanced green fluorescent protein (EGFP) reporter gene, was employed. The plasmids were cultivated in a proficient bacterial strain called Stbl4 and then purified utilizing a DNA extraction kit (FavorPrep™ Taiwan). These purification steps followed the guidelines provided by the manufacturer.

### ZIF-8NP-DNA Complexes Preparation

A combination of ZIF-8NP and CRISPR plasmid DNA (pCAG-eCAS9-GFP-U6-gRNA) was produced. As previously stated, distinct proportions of ZIF-8 were created and exposed to an incubation period of 15 min. Following this duration, the CRISPR vector was introduced into each concentration at 20 ng µL^−1^ and exposed to an additional 30-min incubation period within an incubator. The spermatozoa were subsequently utilized for fertilizing the oocyte.

### Evaluation of the effect of ZIF-8 on Sperm Viability and Motility

To evaluate the viability of the samples, each one was mixed with a 0.67% eosin Y dissolved in 0.9% normal saline)% w/v, acquired from Sigma Aldrich (Germany), in a saline solution. A droplet measuring five μl of this blend was carefully positioned onto a microscope slide and subsequently overlaid with a coverslip, followed by 30 s of equilibration. The samples were then observed under a microscope, with a magnification of 400 × , and a minimum of 200 sperm were counted and classified as either stained (appearing red or dark-pink, indicative of 'dead' sperm) or unstained (appearing white or light-pink, indicative of 'live' sperm). All the concentrations were prepared three times, and the mean of each concentration was considered and compared to the control. The sperm motility analysis was conducted manually, following the guidelines the World Health Organization (WHO) suggested. The sperm motility of each sample was assessed and categorized as progressive, non-progressive, and immotile. After 30 min of incubation,10 ul of sperm samples from each group were loaded onto a microscope slide. Then, the motility was observed under a microscope with a magnification of 400 × .

### Evaluation of acrosome integrity

The acrosomal reaction is a pivotal process that plays a critical role during fertilization in spermatozoa. This intricate series of events involves the fusion of the outer acrosomal membrane with the plasma membrane of the sperm, leading to the release of acrosomal contents. The spermatozoon's binding to the oocyte's zona pellucida initiates this process, which triggers intracellular signaling pathways and raises intracellular calcium levels. To evaluate the acrosomal reaction, the semen was subjected to two washes in PBS through a brief centrifugation process (500 × g, 20 s). It was then fixed in 3.7% paraformaldehyde/PBS for 10 min. The samples were resuspended in PBS and subjected to another brief centrifugation process, after which they were spread onto slides and left to air-dry. A staining process was initiated by introducing 0.22% Coomassie blue G250 to the sperm smear for two minutes, following which it was washed in H2O. The slides were subsequently prepared and evaluated for acrosome-intact spermatozoa, with at least 200 spermatozoa being examined under bright field microscopy. Acrosome-intact spermatozoa stained with Coomassie blue, while the damaged spermatozoa did not exhibit staining.

### ROS Assay assessment

Reactive oxygen species (ROS) levels were assessed using a chemiluminescence assay based on luminol. To perform this assay, spermatozoa that had been exposed to varying concentrations of ZIF-8, briefly, spermatozoa treated in ZIF-8 were gathered and washed three times in PBS, and then the supernatant was collected for each concentration. For each concentration of ZIF-8, 390 μL of ZIF-8 mixed with ten μL of luminol was introduced into the lower section of the tubes and mixed with the rest of the supernatant through vortexing. PBS was utilized as a blank sample (400 μL), while hydrogen peroxide (30% (w/w)) served as the positive control (340 μL of PBS + 50 μL of hydrogen peroxide). Additionally, PBS treated with luminol five mM served as the negative control) 390 μL of PBS + 10 μL of luminol five mM). All labeled tubes were carefully placed in a luminometer within a 96-well plate. Each sample was repeated three times and measured for 15 min. The negative controls, samples, and positive controls were analyzed to calculate the average Relative Luminescence Units (RLU). The ROS level of the samples was determined by dividing it by the sperm concentration mL^−1^.

### Sperm genome extraction

To determine the copy number of plasmid in the sperm nucleolus, it is necessary to extract the sperm genome. To accomplish this, the spermatozoa of mice 10^6^ were subjected to different concentrations of ZIF-8 containing 7.5, 15, 30, and 60 ug mL ^−1^, along with a control group (sperm incubated in HTF). The plasmid concentration used was 20 ng ul^−1(^Plasmid pCAG-eCAS9-GFP-U6-gRNA). The incubation was performed for 30 min at 37.5 °C with 5% (v/v) CO_2_ in the air with high humidity. Following the incubation period, the sperm was retrieved and thoroughly rinsed three times in a phosphate-buffered saline (PBS) solution using centrifugation at 600 g for five minutes. Subsequently,20 U of DNase I (Invitrogen) was applied to the sperm for 30 min to remove exogenous DNA adsorbed on the sperm surface but not internalized. Following the DNaseI treatment, the sperm were washed three additional times in PBS (Ca2 + and Mg2 + free) via centrifugation for 5 min at 600 g, and the sperm pellet was utilized to extract DNA using a genome extraction kit.

### Detection of exogenous DNA by PCR

The sperm genome was extracted in various concentrations of ZIF-8 by extraction kit, as stated above. We designed a primer specifically in our vector construct to determine if the plasmid incubated with spermatozoa exists in the nucleolus. To do this, we used U6 primer as sense and guide antisense.

Primers used in PCR were as follows: AAACTCATTTAGATCCACTGGGCC (guide GDF-8 antisense) and GATACAAGGCTGTTAGAGAG (U6 Universal) (Biogen CO, Germany). The PCR reaction mixture (with a volume of 50 µL) included ten ng genomic DNA, 2.5 mM MgCl2, 250 _M of each dNTP, 10 nM of each primer, and three units of Taq DNA polymerase. The PCR mixture was denatured at 95 C for 5 min, amplified for 40 cycles (95 C° for 30 s, 58 C° for 30 s, and 72 C° for 45 s), and then incubated at 72 C° for 10 min. PCR products were analyzed through electrophoresis using a 2% (w/v) agarose gel.

### Quantification of DNA uptake by quantitative PCR

We have reliably evaluated the quantity of exogenous DNA molecules that were taken up by sperm through the utilization of q-PCR. This technique, which is considered the most effective method for quantifying nucleic acids, has been effectively accomplished for quantifying the uptake of foreign DNA in the spermatozoa of various animal species. Initially, we established calibration curves to determine the exogenous DNA content in our samples, employing the plasmid vector pCAG-eCAS9-GFP-U6-gRNA; the ß-actin gene served as a reference point within the study. To assess its presence, both markers were examined using various diluted forms of either pure plasmid DNA or genomic DNA. It enabled us to determine not only the quantity of exogenous DNA absorbed by sperm cells and transferred into the nucleus but also the amount of the native ß-actin gene present in the identical sample. The method employed allowed for the measurement of DNA uptake efficiency by determining the number of exogenous plasmids absorbed into each sperm nucleus, thereby representing the number of plasmid DNA copies per sperm nucleus.

### Assessment of Fertilization Ability and Early Embryo Development

To investigate the impact of ZIF-8 on sperm fertility and the development of early embryos at the blastocysts stage, a series of in vitro fertilization assays were conducted. To accomplish this goal, we employed eight to ten-week-old B6D2F1 mice. These mice were given a superovulation treatment consisting of 10 IU of pregnant mare serum gonadotropin (PMSG), followed by an injection of 10 IU of human chorionic gonadotropin (HCG) 50 h later. We collected Metaphase II oocytes from the oviduct ampulla 14–16 h after the HCG injection. These oocytes and their cumulus complexes were released into a flushing holding medium (FHM). Afterward, we placed them in 100 μl of Human Tubal Fluid (HTF) drops and covered them with mineral oil.

To evaluate the effects of ZIF-8, we subjected sperm cells to incubation under capacitating conditions using various concentrations of ZIF-8. Once the sperm cells were incubated for 30 min, a high concentration (0.5 × 107 motile sperm cells/ml) was added to the oocyte petri dish for fertilization. Additionally, we added HTF groups to separate drops containing the oocyte medium, which were then placed in an incubator. In the following 5–6 h after in vitro fertilization (IVF), the oocytes' cultures were moved into KSOM, an enriched medium containing both nonessential and essential amino acids, 4 mg/ml bovine serum albumin (BSA), and stored at a temperature of 37 °C with a 5% CO2 environment. The growth and development of the embryos until they reached the blastocyst stage (96 h after fertilization), were examined in each group. Furthermore, the morphology of the blastocysts was recorded 96 h after fertilization, and the expression of EGFP (Enhanced Green Fluorescent Protein) was observed using inverted microscopy.

### Statistical Analysis

The data presented in this research is conveyed as mean, standard error. Comparisons in different groups were performed through the one-way ANOVA, considering a significant *P*-value of < 0.05. The statistical analysis was conducted using the SPSS19 software (Somers, NY) and graphically represented using GraphPad Prism 9.5.1, developed by GraphPad Software in San Diego, CA.

## Results

### Crystal structure and morphology characteristic

**Fig. 2:**
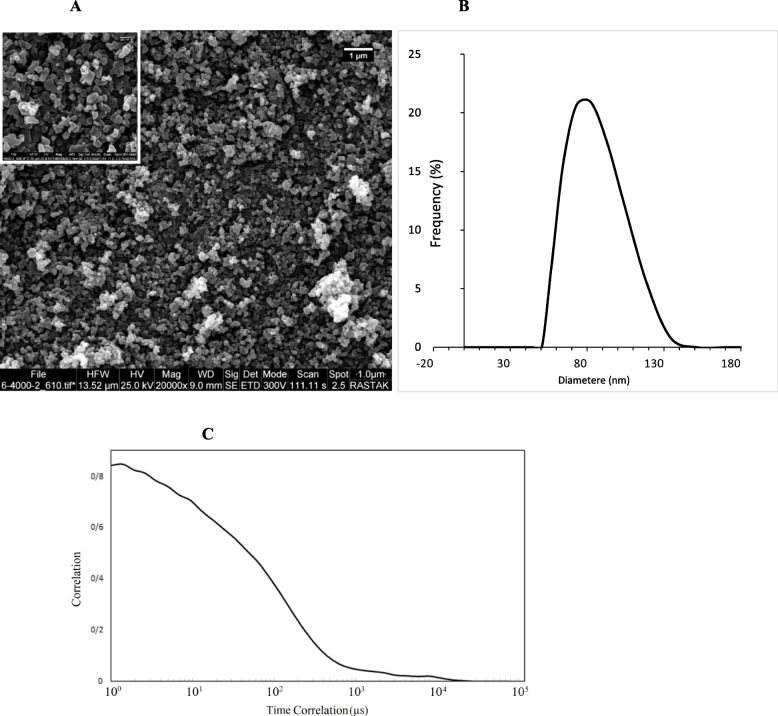
Characterization of ZIF-8 particle size composite using **a** FESEM image of ZIF-8, and **b** Dynamic Light Scattering (DLS) (Scale bars represent 1 µm). **c** Autocorrelation function of ZIF-8NP

**Fig. 3:**
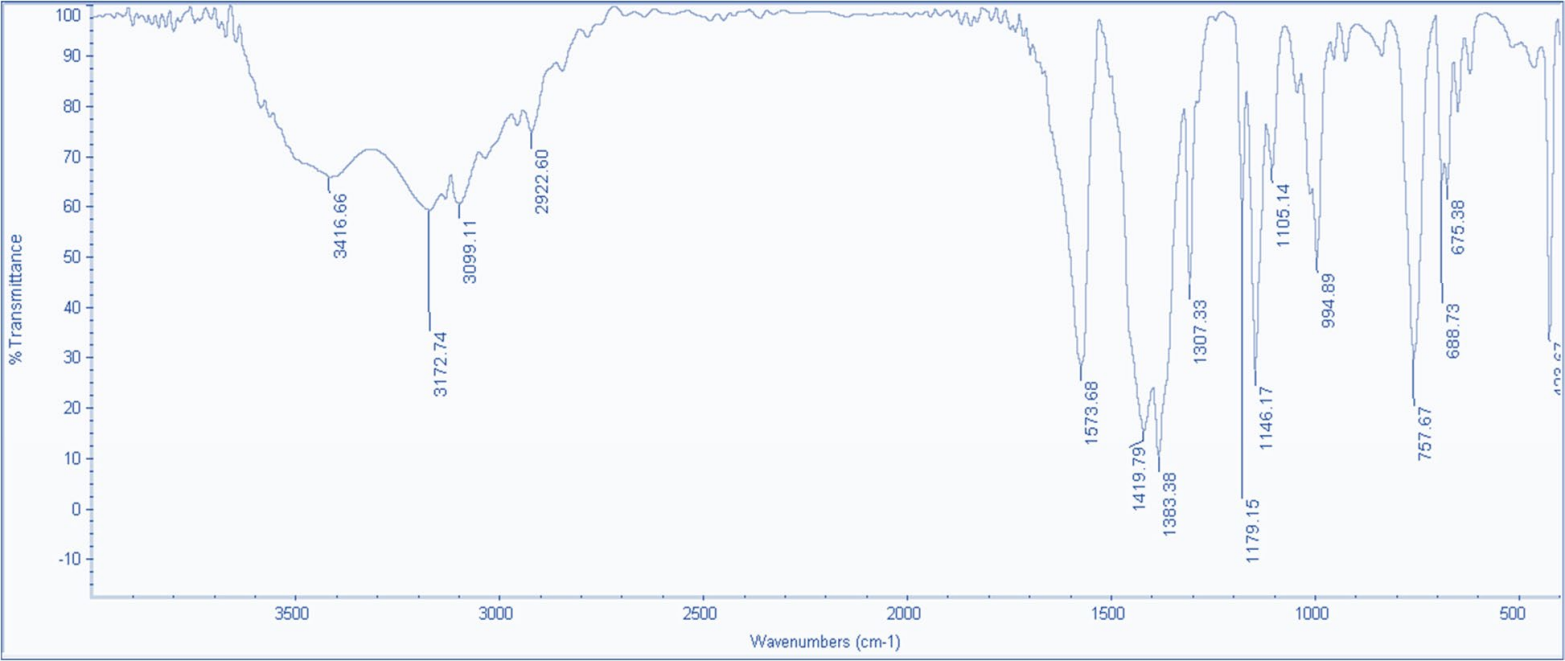
Analyzing the chemical composition and molecular structure of ZIF-8 using FT-IR (Fourier Transform Infrared Spectroscopy)

The synthesized sample was imaged using SEM at two magnifications. As shown in Fig. 2a, formed particles have exhibited well-defined hexagonal morphologies [45, 46]. With uniform size, based on the DLS images, it is evident that the ZIF-8 nanoparticles have an average hydrodynamic particle size of 77.8 nm through hydrodynamic light scattering (Fig. 2b). And also the auto correlation functions of ZIF-8 obtained from DLS measurements (Fig. 2c). As depicted in Fig. 3, the FTIR results showed the spectra of the synthesized ZIF-8 sample peaks at 423 cm^−1^, which related to the Zn-N bond, and peaks at 1422.8 cm^−1^ and 1578 cm^−1^ related to C–C and C = N bonds, respectively. Additionally, peaks at 1578 cm^−1^ and 2932 cm^−1^ have been attributed to aliphatic C-H and aromatic C-H, respectively. The spectra within the range of 600–1500 cm^−1^ have indicated bending and stretching modes in the imidazole ring [47, 48].

**Fig. 4:**
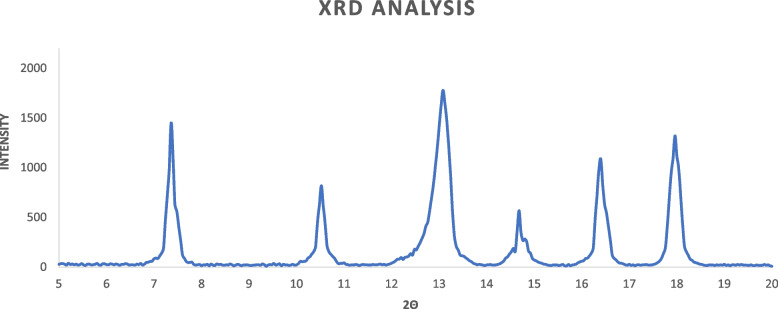
XRD diffraction patterns of the synthesized ZIF-8. The diffraction peaks indicate the presence of specific crystal planes corresponding to the ZIF-8 framework. The diffraction angle (2θ) is plotted on the x-axis, while the intensity is plotted on the y-axis. The distinct diffraction peaks observed at specific angles confirm the successful synthesis of ZIF-8 with well-defined crystallinity

Thus, the findings from FT-IR analysis have provided evidence of the effective coordination between metal clusters and organic linkers within the framework. The validity of the crystal's composition was also confirmed through X-ray diffraction patterns (XRD) in Fig. 4. These XRD spectra have exhibited distinct and well-defined peaks, which strongly align with the expected sodalite structure as simulated. The presence of such sharp peaks serves as an indication of the exceptional level of crystallinity observed in the synthesized ZIF-8 nanoparticles. Specifically, the 2θ peaks at 7.30, 10.35, 12.7, 14.8, 16.4, and 18 correspond to the crystallographic orientations 110, 200, 211, 220, 310, and 222, respectively.

### Effect of ZIF-8 on Spermatozoa Viability, Motility, and Acrosomal Reaction

We investigated the impact of ZIF-8 on the viability, motility, and acrosomal integrity of sperm. As shown in Table 1, the results obtained by eosin Y staining showed that the viability of spermatozoa was 87 ± 1.73, 86.33 ± 1.20, 85.33 ± 0.66and 85 ± 1.52 after incubation with 7.5, 15, 30, 60, 90, 120 and μg mL^−1^ ZIF-8, respectively. The viability of spermatozoa did not differ significantly between groups compared to control. However, there was a significant difference in the viability of the spermatozoa between the groups and DMSO (3%). We also evaluated the viability of spermatozoa when treated with ZIF-8NP at concentrations of 7.5, 15, 30, and 60 μg mL^−1^ with plasmid DNA, as presented in Table 1. We assessed the progressive motility for varying concentrations of ZIF-8. The progressive movement of the DMSO group is significantly associated with all concentrations of ZIF-8. Furthermore, as demonstrated in Table 1, there are significant differences observed between the groups. We also evaluated the acrosomal reaction, a pivotal process that plays a critical role during fertilization in spermatozoa. As indicated in Table 1, the acrosomal reaction had no significant difference between groups compared to the control.

**Table 1:** Sperm characteristics determined in male mice while treated with ZIF-8NP and after exposure to plasmid CRISPR/CAS9 (mean ± SEM)

	**Viability (%)**	**Acrosomal reaction (%)**	**Progressive Motility (%)**
Control	87.33 ± 1.45 ^a^	4.33 ± 0.33 ^a^	95.00 ± 0.57 ^a^
DMSO + Plasmid	43.00 ± 0.55 ^b^	21.03 ± 0.27 ^b^	7.66 ± 1.45 ^b^
7.5 µg ml^−1^	87 ± 1.73 ^a^	4.00 ± 0.57 ^a^	84.66 ± 0.33 ^a,c,d,e^
15 µg ml^−1^	86.33 ± 1.20 ^a^	3.33 ± 0.33 ^a^	74.66 ± 0.33 ^c,d,e^
30 µg ml^−1^	85.33 ± 0.66 ^a^	3.33 ± 0.33 ^a^	84.00 ± 0.57 ^a,c,d,e^
60 µg ml^−1^	85 ± 1.52 ^a^	4.00 ± 0.57 ^a^	79.66 ± 0.57 ^d,c,e^
7.5 µg ml^−1^ + Plasmid CAS9	87.33 ± 0.33 ^a^	3.333 ± 0.33 ^a^	87.4 ± 0.4 ^a^
15 µg ml^−1^ + Plasmid CAS9	85.66 ± 0.66 ^a^	4.00 ± 0.57 ^a^	75 ± 0.61 ^d,c,e^
30 µg ml^−1^ + Plasmid CAS9	84.33 ± 0.66 ^a^	4.33 ± 0.33 ^a^	85 ± 0.27 ^a,c,d,e^
60 µg ml^−1^ + Plasmid CAS9	84.00 ± 1.52 ^a^	4.66 ± 0.33 ^a^	78.2 ± 0.5 ^e,c,d^

Values with different alphabetical Superscripts differ significantly (*P* < 0.05)

### ROS distribution by group

The chemiluminescence assay is a widely used approach for measuring reactive oxygen species (ROS) levels in semen samples due to its high sensitivity, specificity, and reproducibility [49].

Moreover, this approach can anticipate spermatozoa's fertility potential since elevated ROS production is associated with sperm dysfunction [50].

**Fig. 5:**
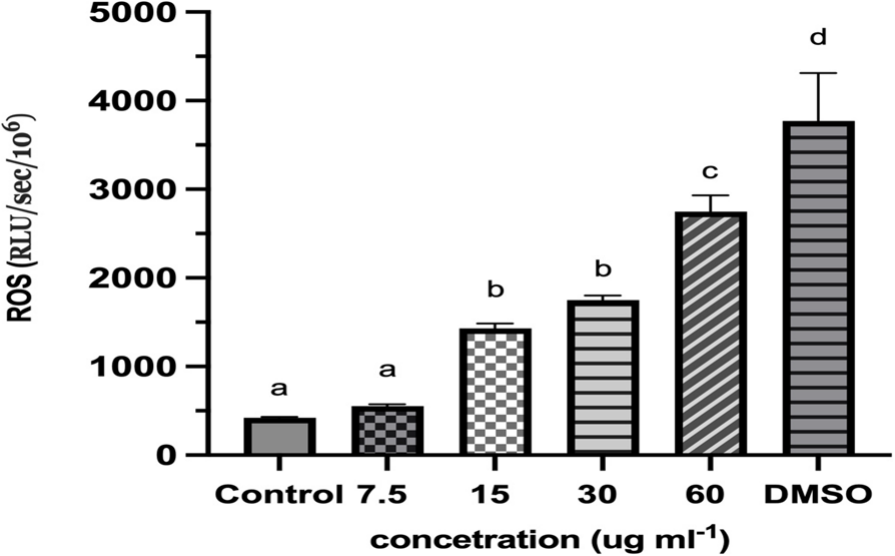
Evaluation of ROS (RLU/s/ × 10^6 ^sperm) between different concertation of ZIF-8 and DMSO. Significant (*p* < 0.05) versus control. The grouped data points in the plot are represented by different alphabetical letters (a, b, c, d) to indicate the significancy between the groups

Figure 5 illustrates the distribution of ROS levels in various concentrations of ZIF-8 compared to DMSO (3%) and a control. The results indicate no significant difference in ROS levels between the control and concentration of 7.5 μg mL^−1^. However, significant differences were observed in the ROS levels of other concentrations of ZIF-8. While no significant difference was observed in the relative light unit (RLU)values for ZIF-8 at 15 μg mL^−1^ and 30 μg mL^−1^, 60 μg mL^−1^ exhibited a significant difference compared to the other concentrations. As shown in this figure, when the ZIF-8 concentration increases, the ROS distribution level also increases as expected. RLU value for each sample was measured in triplicate for 15 min, the mean of every sample was calculated, and a threshold of P < 0.05 was considered for the significance level. We also evaluated the ROS level of blanks, negative control samples, and positive control samples in duplicate. The results obtained from the blank and negative control samples consistently displayed relatively low average Relative Light Units (RLU) values.

In contrast, the positive control samples consistently exhibited high average RLU values, as shown in Supplementary Table S1. No significant disparity was observed in RLU values between the control samples and blanks. As anticipated, RLU (Relative Light Units) values should be relatively low for the test to operate efficiently for both blank and control samples. Conversely, positive control samples should yield a relatively high reading. Blanks are used to measure the background luminescence produced by the luminometer, while control samples provide insight into both background luminescence and impurities in the reagents. Ideally, these values should be similar. Blanks and negative control samples exhibit minimal chemiluminescence, as indicated in Supplementary Table S1, whereas positive control samples display a pronounced chemiluminescence signal, as shown in Table S1.

### Quantitation of Exogenous DNA Internalization into Mice Sperm

**Fig. 6:**
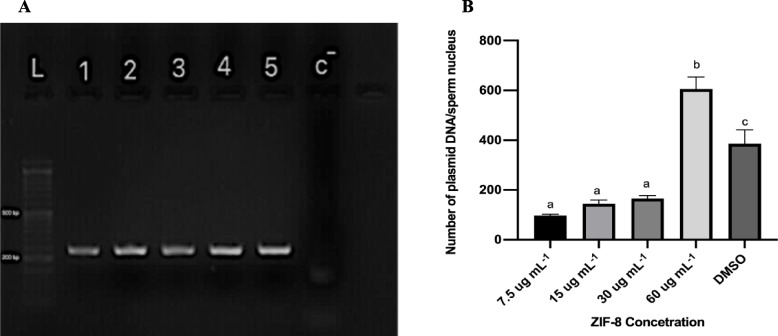
Impact of Different ZIF-8 Concentrations on DNA Uptake Efficiency by Mice Sperm **b**. effect of sperm treatment with various concentrations of ZIF-8 on the efficiency of DNA uptake by mice sperm. Sperm cells were pre-incubated with incubation buffer (CTRL) and 7.5, 15, 30, 60(ug mL^−1^) and DMSO (3%) for 30 min; after DNA incubation, total DNA was extracted from purified nuclei then, the extracted DNA was analyzed via q-PCR, in accordance with the methodology outlined in the Materials and Methods Section Data are means ± standard deviation of at least two independent experiments performed. *P* < 0.05 **a**. Agarose gel electrophoresis of exogenous DNA genomic and ZIF-8NP-DNA complexes: Lanes 1–4, ZIF-8NP/DNA complexes at the concentration of 7.5, 15, 30 and 60 ug ml^−1^ respectively; Lane 5 represents positive control, lane c^−^ (negative control); L: DNA ladder

Initially, PCR genotyping was carried out to determine the plasmid construct in the sperm nucleolus of mice. To do this, we first designed the specific primer for the vector construct and observed the specific band on a 2% agarose gel by electrophoresis, as depicted in Fig. 6(a). The band observed corresponds to an amplification fragment associated with the U6 promoter. The guide designed for the cas9 scaffold was visible in spermatozoa treated with four concentrations of the ZIF-8NP. At the same time, no targeted fragment was shown in sperm treated with only ZIF-8 without vector (negative control) or in the wild-type control.

We reliably employed quantitative polymerase chain reaction (q-PCR) to evaluate the quantity of exogenous DNA molecules taken up by sperm. This method is considered the most effective technique for quantifying nucleic acids and has been successfully validated for measuring the uptake of foreign DNA in spermatozoa from various animal species [12, 51, 52]. Initially, we created calibration curves to determine the concentration of exogenous DNA in samples. To achieve this, we utilized a plasmid vector called pCAG-eCAS9-GFP-U6-gRNA and the β-actin gene serving as our internal control. We thoroughly analyzed the markers by conducting serial dilutions of pure plasmid or genomic DNA. We were able to assess the quantity of exogenous DNA molecules that were taken up into the nucleus of sperm cells, as well as the quantity of native β-actin genes within the same sample. It allowed us to determine the count of exogenous plasmids internalized per sperm nucleus, representing the copies of plasmid DNA in each nucleus.

Consequently, we were able to express the efficiency of DNA uptake. During an initial series of experiments, we fine-tuned the quantity of foreign DNA and the duration and temperature of the incubation process to achieve the maximum number of sperm cells containing DNA without resulting in excessive accumulation. As shown in Fig. 6(b), Upon thorough investigation, it was found that treatment with ZIF-8 significantly increased the number of copies of plasmid vector in each sperm nucleus; specifically, ZIF-8 enhanced the effectiveness of DNA uptake by 60 ug mL-1 ZIF-8.

### Spermatozoa Fertilizing Ability and Early Embryo Development

Finally, we examined the potential of ZIF-8-treated sperm cells to fertilize eggs and undergo normal embryo development effectively. To achieve this, we conducted a fertilization process by combining in vitro matured oocytes with in vitro capacitated sperm cells. This procedure was carried out under two sets of conditions: the standard control conditions and an additional treatment involving the utilization of ZIF-8 and ZIF-8-DNA.As summarized in Table 2, we observed no significant differences in the fertilization rates between the different concentrations of ZIF-8 and control groups. Furthermore, the control group successfully retained its capability to fertilize oocytes in vitro.

**Table 2:** Effect of ZIF-8 on mouse embryo development after in vitro fertilization

	**Oocyte no**^**o**^	**Fertilization (%)**	**Four cell Stage (%)**	**Compact (%)**	**Blastocyst (%)**	**GDF Positive (%)**
Control	96	89.05 ± 2.07 ^a^	89.05 ± 2.07 ^a^	87.21 ± 2.71^a^	86.56 ± 1.09 ^a^	ND
DMSO + Plasmid	89	85.00 ± 5.09 ^a^	85.00 ± 5.09 ^a^	83.04 ± 2.50 ^a^	81.09 ± 0.47 ^a^	44.09 ± 1.64 ^a^
7.5 µg ml^−1^	83	95.48 ± 1.10 ^a^	85.67 ± 1.98 ^a^	82.70 ± 2.58 ^a^	81.45 ± 4.57^a^	ND
15 µg ml^−1^	66	92.85 ± 2.12 ^a^	92.85 ± 3.68 ^a^	92.85 ± 2.12 ^a^	90.36 ± 3.88 ^a^	ND
30 µg ml^−1^	86	92.59 ± 4.90 ^a^	83.62 ± 0.50 ^a^	83.62 ± 0.29 ^a^	83.62 ± 0.59 ^a^	ND
60 µg ml^−1^	99	93.93 ± 5.33 ^a^	92.31 ± 6.65 ^a^	84.83 ± 0.31 ^a^	83.33 ± 0.88 ^a^	ND
7.5 µg ml^−1^ + Plasmid CAS9	65	91.87 ± 4.27 ^a^	86.58 ± 1.29 ^a^	83.27 ± 2.29 ^a^	82.15 ± 0.85 ^a^	58.87 ± 3.32 ^a^
15 µg ml^−1^ + Plasmid CAS9	73	92.85 ± 2.12 ^a^	92.85 ± 2.12 ^a^	92.86 ± 2.12 ^a^	89.66 ± 2.40 ^a^	76.38 ± 3.00 ^b^
30 µg ml^−1^ + Plasmid CAS9	86	92.59 ± 4.90 ^a^	83.55 ± 0.33 ^a^	83.55 ± 0.33 ^a^	81.18 ± 3.11 ^a^	83.61 ± 5.19 ^b^
60 µg ml^−1^ + Plasmid CAS9	66	94.04 ± 3.01 ^a^	87.08 ± 0.43 ^a^	85.08 ± 0.43 ^a^	85.08 ± 0.58 ^a^	84.96 ± 2.29 ^b^

The rates of different stages were calculated by dividing the average values of each stage by the number of two pronuclear (2PN) stages. To determine the fertilization rate, the number of (2PN) cells was divided by the total number of oocytes. (Data are shown as number means ± SEM). (*P* < 0.05). Significant differences in the same column are indicated by different superscript letters, a, b

**Fig. 7:**
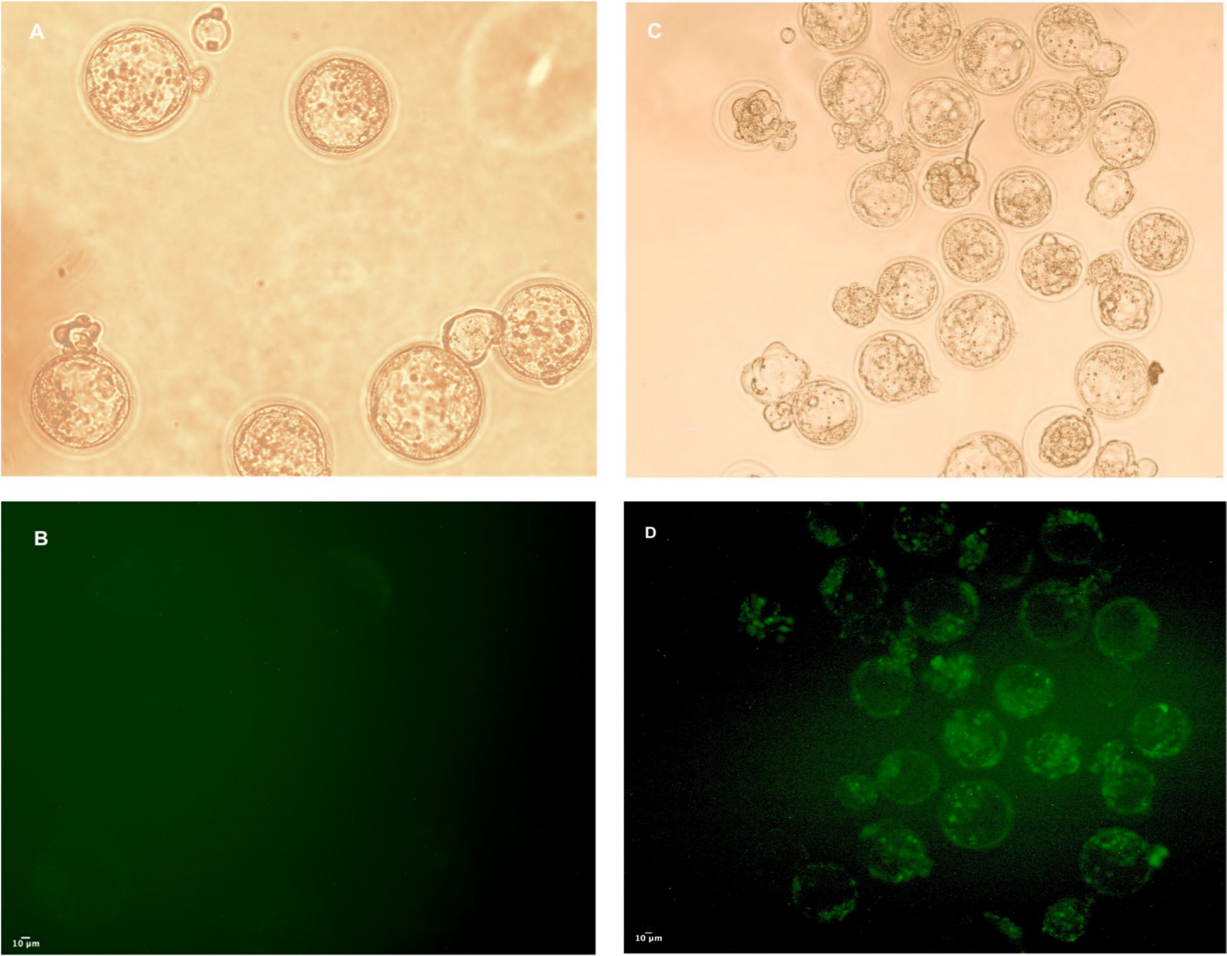
Mouse blastocysts in treatment with ZIF-8, compared with control. Sperm incubated with EGFP plasmid, Cleaved, and blastocyst embryos under bright field; A and C: The embryos expressing the EGFP gene shown under blue light D; Original magnification 200x, scale bar 10 μm

As shown in Table 2, the subsequent cleavage stages demonstrated no significant differences between groups in the 4-cell, compact cell, and blastocyst stages. However, the transfection rate, which is the ability of DNA uptake by ZIF-8, was significantly higher at a concentration of 60 μg mL^−1^ with a rate of 84.96 ± 2.29, indicating its more remarkable ability to transfect the blastocysts. In Fig. 7, we presented the ability of ZIF-8 to produce transgenic blastocysts following in vitro development. The control group did not show any GDF-positive blastocysts, whereas the green blastocyst confirmed the transgenic blastocysts produced by ZIF-8.

## Discussion

There has been an increasing trend in recent times to explore various nanotechnology instruments for reproductive biology with the primary objective being the improvement of the targeted and controlled delivery of different substances into reproductive tissues, gametes, and embryos. This advancement holds particular value from a reproductive biology perspective for experimental research, diagnostics, and therapeutic purposes [53]. Nanomaterials have gained widespread recognition for their ability to enhance the absorption of external substances into cells. Interestingly, reproductive tissues and gametes, especially sperm, possess unique characteristics that naturally impede the uptake of molecular cargo at significant physiological levels [54]; one of the challenges encountered in research methods involving the transport of investigative compounds into cells is the potential limitation it imposes on performance. Currently, the primary approach utilized to enhance the entry of compounds into sperm cells is through artificial permeabilization of the cell membranes using detergents. However, this technique can be aggressive and may adversely affect the structural integrity of DNA and the developmental capacity of gamete [55, 56]; Furthermore, it is unsuitable for experiments investigating the intricate mechanisms of fertilization and subsequent embryo development [57].

The primary objective of this study was to explore the possibility of utilizing ZIF-8-mediated gene delivery to facilitate the introduction of exogenous DNA into sperm cells, aiming to generate transgenic blastocysts through a novel approach. The limited success rate of the SMGT technique can primarily be attributed to the inadequate uptake of exogenous DNA by sperm cells [7].

This study successfully illustrated the ideal circumstances for the entry of exogenous DNA to mouse spermatozoa using ZIF-8. Our findings unequivocally show that the ZIF-8NP can effectively transport exogenous DNA into the sperm cells of mice. Furthermore, we discovered that the ZIF-8NP exhibits a remarkable capability to generate transgenic blastocysts, which holds great potential as a revolutionary approach for generating transgenic animals. The development of targeted nanotherapeutics that can reach the external cell membrane and enhance uptake [58–60] necessitates organism-based investigations to evaluate their safety and viability. To our knowledge, this is the first study addressing the use of ZIF-8NP in mammalian sperm. Our initial focus was to investigate the impact of ZIF-8NP on sperm viability and ensure its non-toxic effects on the cells.

Based on our investigations, it has been observed that the presence of ZIF-8NPs in the media did not exhibit any detrimental effects on the survival of spermatozoa and maintains cell viability at an excellent level, suggesting that ZIF-8NP is a non-cytotoxic means for sperm cells. To validate our results, many research investigations have been carried out to evaluate the cytotoxicity of ZIF-8 in various cell lines. For instance, Kamal et al. demonstrated that pure ZIF-8 exhibited the lowest toxicity in terms of survival rate when incubated with embryos for 24 to 96 h.

 [58]. We also exposed spermatozoa to high doses of ZIF-8, such as 90, 120, 150, and 200 μg mL^−1^, which reduced viability without significant differences. However, embryo development was halted at different stages, indicating that high doses of ZIF-8 did not affect spermatozoa viability but could arrest embryo development. Thus, we can conclude that the cytotoxicity of ZIF-8 is dose-dependent, and numerous studies have evaluated the optimal ZIF-8 dose. For example, Hoop et al. investigated the influence of different concentrations of ZIF-8 (ranging from 0 to 100 μg mL-1) on the viability and growth of six different types of cells. Their findings revealed that ZIF-8 concentrations up to 30 μg mL-1 caused only a slight decline in cell viability, reducing it to approximately 80% compared to the control group [61]. Furthermore, Shahad and his colleagues embarked on a comprehensive investigation to determine the lethal dose (LD50) of ZIF-8 in the CHO cell line, which was found to be 400 μg mL-1. Additionally, ZIF-8 concentrations equal to or greater than 200 μg mL-1 were observed to increase cytotoxicity [62].

In addition, apart from the natural toxicity associated with ZIF-8, there is also the possibility of encountering toxicity issues arising from the methods used for its synthesis. Specifically, this pertains to the procedures that utilize solvents like N, N-dimethylformamide (DMF), and reaction modulators in the preparation of nanoparticles. Residual solvents may pose potential toxicity issues. In our investigation, we used a water-based synthesis method for ZIF-8, a simple and environmentally friendly approach that reduces the toxicity of ZIF-8 in various cell types. The effectiveness of spermatozoa exposed to ZIF-8 can be attributed to either one or both elements in ZIF-8 crystals (Zn2 + or 2-mIM). A comprehensive examination of the constituent 2-mIM in a study conducted on rats concluded that it does not exhibit any noteworthy toxicological impact [63]. In conclusion, the cytotoxicity of ZIF-8 is contingent on several factors, including particle size, synthesis method, and dosage.

Another critical factor correlating to sperm fertilization capacity is sperm motility, which is regulated by several metabolic pathways and regulatory mechanisms [64]. Our findings demonstrate that treatment with ZIF-8NP affects sperm motility in a dose-dependent manner, with increased doses resulting in reduced motility. Since ZIF-8NP consists of two primary particles (Zn2 + and 2-mIM), these components may impact sperm motility. Numerous studies have assessed the impact of zinc dosage on sperm motility. In a study conducted by Ahmed et al., it was found that zinc plays a crucial role in spermatozoa's motility by controlling energy utilization in the ATP system. This system contributes to muscle contraction and regulates the energy reserves stored in phospholipids. Remarkably, zinc ions are firmly attached to the surface of sperm cells, as proven by numerous investigations [65].

Studies have indicated that zinc deficiency in rats can impair spermatogenesis and atrophy of the seminiferous tubule [66]. The absence of zinc in the seminal plasma can disrupt DNA stability, accelerate the acrosome reaction process, and reduce ATP levels [67]. However, several studies have reported that high zinc levels may decrease sperm motility. Additionally, there have been reports indicating that elevated levels of zinc in the seminal plasma or sperm tails harm the movement and mobility of sperm [68, 69]. Several studies have used zinc as a component of nanoparticles to assess its role in sperm characterization [70–73]. These studies have further demonstrated that the harmful effects of ZnO on sperm cells rely solely on the quantity and duration of the substance's presence [74]. As there is no prior research on the impact of ZIF-8NP on mammalian sperm cells, we can infer that the effect of ZIF-8NP on sperm motility is dose-dependent, with an increase in dosage resulting in decreased motility. Based on this observation, we can hypothesize that as the nanoparticle dosage increases, there is a corresponding increase in zinc plasma concentrations. It may have a detrimental effect on spermatozoa motility.

Reactive oxygen species (ROS) are pivotal in a diverse range of cellular signaling mechanisms, and their interaction with lipids, proteins, and DNA can give rise to severe pathological disorders. Increased ROS levels have detrimental effects on gametes, impairing their functionality through the initiation of lipid peroxidation, protein impairment, and DNA strand damage. While ROS production is a physiological process in spermatozoa to facilitate sperm capacitation, certain pathological conditions can disrupt the balance between ROS levels and sperm antioxidant defenses in semen, leading to oxidative stress that negates successful fertilization and hampers embryonic development. Our findings indicate that as the concentration of ZIF-8 increases, the ROS assay value also increases. However, at a 60 μg mL^−1^ concentration, an increase in ROS levels did not affect embryo development.

Nevertheless, concentrations above 60 μg mL^−1^, such as 90 and 120 μg mL^−1^, arrested in vitro fertilization at various stages, which could be attributed to increased ROS levels. Multiple research studies have established that heightened levels of ROS in seminal fluids can overcome the antioxidant defenses of sperm, resulting in oxidative stress. This oxidative stress not only hampers the fertilization process but also adversely affects the development of embryos [75].

As stated above, ZIF-8 comprises two primary components that could significantly impact elevated ROS levels. One of these components is zinc. Several studies have suggested that zinc deficiency could negatively impact sperm function. Conversely, increased intracellular levels of zinc ions could harm sperm cells by promoting ROS production. Higher levels of Zn2 + inside cells have been found to obstruct enzymes linked to the Krebs cycle, such as glycerol-3-phosphate dehydrogenase. This elevated concentration of Zn2 + also triggers the permeability transition of the mitochondrial membrane and hampers the mitochondrial bc1 cytochrome complex.

Consequently, this leads to a more significant generation of ROS buildup [76–79]. Previous studies by Yu et al., and Horcajada et al. have reported an augmentation in the production of re ROS by MOFs incorporating metallic nodes like iron (Fe), chromium (Cr), and aluminum (Al) ions [80–82]. As a result, we predicted that more ZIF-8 crystals in the sperm media would increase Zn2 + levels. It, in turn, would lead to an elevation of intracellular ROS levels due to the effects mentioned above. One proposed theory suggests that ROS hinders the function of specific enzymes, such as glucose-6-phosphate dehydrogenase (G6PD). This enzyme plays a crucial role in regulating the accessibility of NADPH within the cells through the hexose monophosphate pathway. The inhibition of G6PD enzyme activity causes a reduction in the presence of NADPH and a simultaneous buildup of both oxidized and reduced forms of glutathione. Consequently, the sperm's defense against harmful substances is compromised, leading to the occurrence of peroxidation in the phospholipids of the cell membrane [83, 84]. Another way injury may occur is elevated levels of ROS, triggering a series of interconnected events that lead to a decline in the axoneme protein phosphorylation. Ultimately, this results in the sperm immobilization of [85]. The effects of ROS on sperm function are being used to develop contraceptives.

Exogenous DNA delivery into sperm cells is a significant challenge in nanoSMGT for generating transgenic animals, and various studies have explored the use of nano-carriers to deliver DNA into spermatozoa. Previous research using different cell lines has suggested that endosomal escape is the primary mechanism for ZIF-8NP entry [43, 86–92]. Our findings demonstrate that ZIF-8NPs are potential gene nano-carriers for mice sperm cells. Due to its size (< 100 nm), it can easily penetrate the cell membrane. In a study by Alsaiari et al., it has been reported that ZIF-8 can protect both negatively charged sgRNA and substantial Cas9 proteins. Furthermore, ZIF-8 exhibits a remarkable loading capacity of 1.2% (w/w).

To achieve successful genetic transfection, it is crucial to protonate the imidazole-based framework at the endosomal pH, facilitating rapid endosomal escape and enhanced nuclear delivery. Successful endosomal escape may be attributed to the proton sponge effect of the imidazole ring in ZIF-8, which promotes endosomal escape [62]. This research aims to investigate the impact of ZIF-8 on DNA uptake by mouse spermatozoa. Therefore, we examined the DNA uptake ability of spermatozoa at various concentrations of ZIF-8NPs. Our qPCR results revealed that the highest DNA uptake by spermatozoa was observed at a concentration of 60 μg mL^−1^ without any detrimental effects on motility and viability. Although the ROS level increased, it did not harm embryo development. In a previous study where different cell lines were treated with ZIF-8, the optimal dosage was determined to be 30 μg mL^−1^ to achieve the best results. As there was no prior research on using ZIF-8 to treat mammalian sperm, our study demonstrated that 60 μg mL^−1^ concentration is the optimal dosage for generating transgenic blastocysts.

## Conclusions

In this research, we successfully introduced the EGFP gene into mouse sperm cells and subsequently generated transgenic blastocysts using SMGT. Our findings demonstrate that forming ZIF-8NP-DNA complexes between ZIF-8 nanoparticles and plasmid DNA enable the uptake of exogenous DNA by mouse sperm cells. We then utilized IVF to transfer the introduced DNA into oocytes, producing transgenic blastocysts. Through qPCR analysis, we determined that increasing the copy number of plasmid pCAG-eCAS9-GFP-U6-gRNA enhances the efficiency of SMGT. Based on our results, ZIF-8NP-mediated SMGT shows excellent promise as a novel technique for generating transgenic animals.

### Supplementary Information

**Additional file 1:** **Supplementary Table S1.** RLU values between the control samples and blanks and psitive control. **Supplementary Table S2.** The autocorrelation data of DLS. **Supplementary Table S3.** Dynamic Light Scattering (DLS). **Supplementary Table S4.** Zeta potential distribution of ZIF-8.

**Additional file 2. Figure S1:** SEM image of ZIF-8 after sonication for 30 minutes. **Figure S2:** DLS measurement of ZIF-8. **Figure S3:** Zeta Potential of ZIF-8.

## Data Availability

All data generated or analyzed during this study are included in this published article.
