# Supplementary material for: ZIF-8 Nanoparticle: A Valuable Tool for Improving Gene Delivery in Sperm-Mediated Gene Transfer
Source: Biol Proced Online. 2024 Jan 26;26:4. doi: 10.1186/s12575-024-00229-2 (PMC10811821; doi:10.1186/s12575-024-00229-2)
Supplement: Supplementary file 2 — Additional file 2. Figure S1: SEM image of ZIF-8 after sonication for 30 minutes. Figure S2: DLS measurement of ZIF-8. Figure S3: Zeta Potential of ZIF-8. [file 12575_2024_229_MOESM2_ESM.docx]

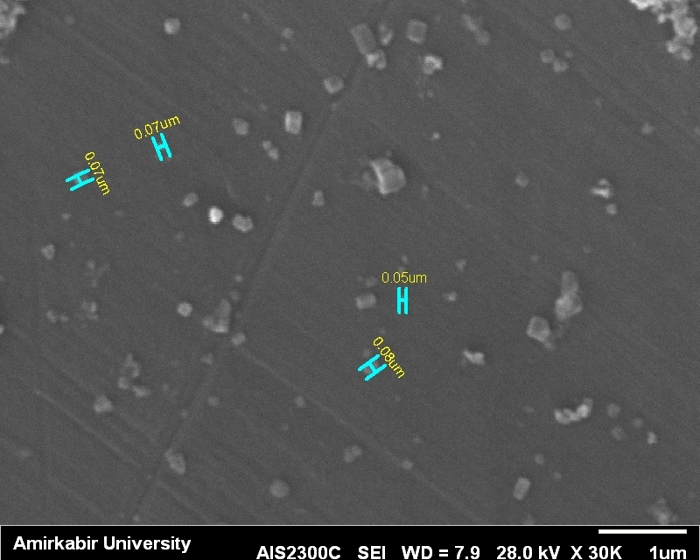


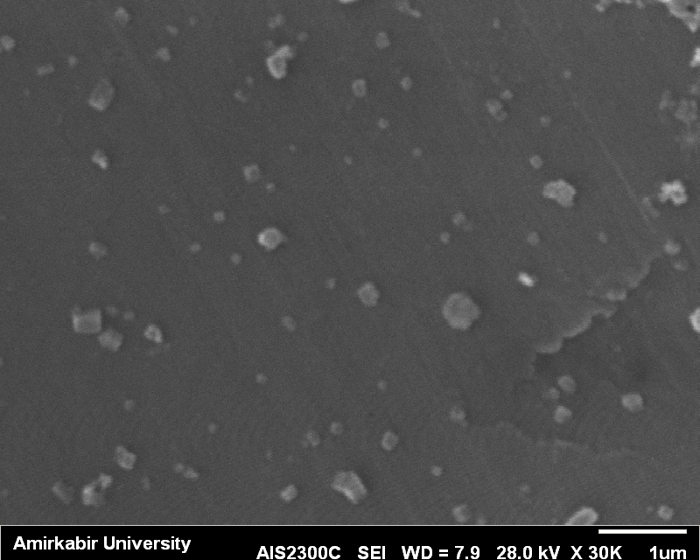


Figure S1


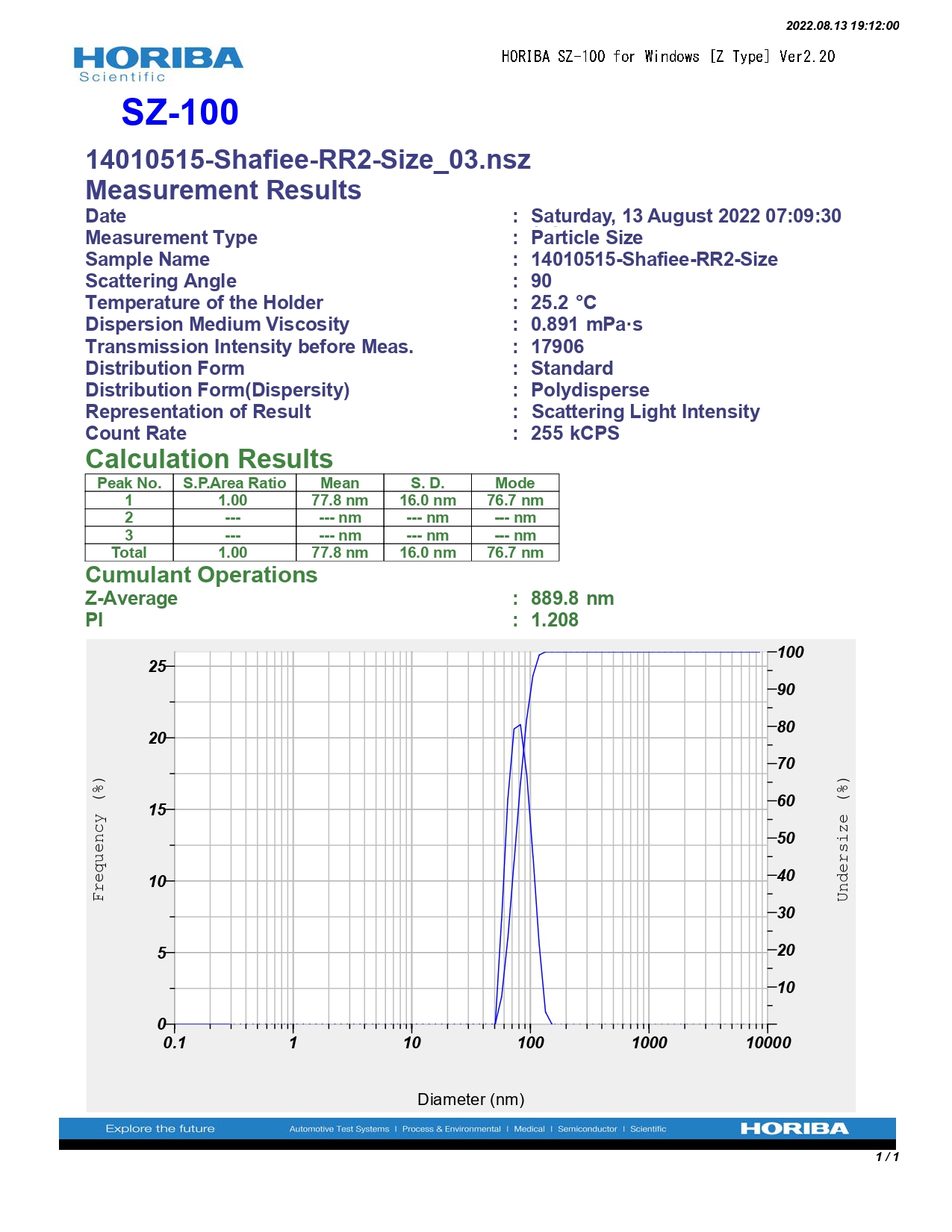


Figure S2


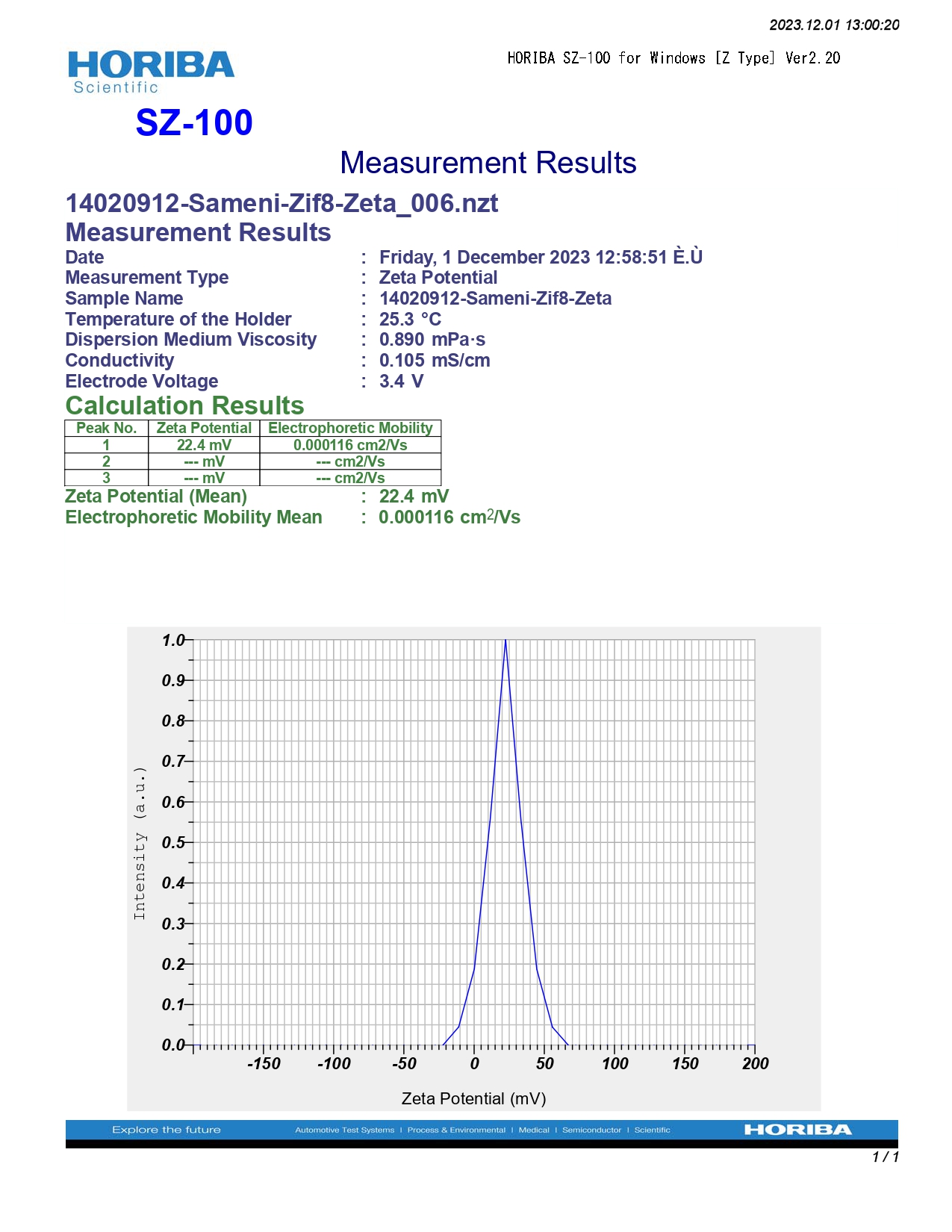


Figure S3

Figure S1: SEM image of ZIF-8 after sonication for 30 minutes

Figure S2: DLS measurement of ZIF-8

Figure S3: Zeta Potential of ZIF-8
